# Suppression of Sebum Production by Vemurafenib Through Paradoxical ERK Activation Resulted in the Inhibition of the mTOR Pathway in 5α‐Dihydrotestosterone‐Differentiated Hamster Sebocytes In Vitro

**DOI:** 10.1111/exd.70150

**Published:** 2025-08-16

**Authors:** Toshikazu Koiwai, Takashi Sato

**Affiliations:** ^1^ Department of Biochemistry Tokyo University of Pharmacy and Life Sciences Tokyo Japan

**Keywords:** 5α‐dihydrotestosterone, extracellular signal‐regulated kinase, mechanistic target of rapamycin, sebaceous glands, vemurafenib

## Abstract

Vemurafenib, a low‐molecular‐weight BRAF inhibitor, effectively treats cutaneous melanoma with the BRAF^V600E^ mutation, but it causes skin disorders such as dry skin with high frequency. As one of the factors causing skin dryness is a decrease in sebum production due to sebaceous gland dysfunction, we examined whether vemurafenib regulated the production and accumulation of sebum in hamster sebocytes. Vemurafenib dose‐ and time‐dependently decreased the production and accumulation of sebum in DHT‐differentiated hamster sebocytes. In addition, the DHT‐augmented gene expressions of SCD‐1, DGAT‐1 and PLIN‐1, which are involved in sebum production and accumulation in sebaceous glands, were suppressed by vemurafenib in hamster sebocytes. Unexpectedly, vemurafenib facilitated ERK phosphorylation in hamster sebocytes. In addition, DHT augmented the phosphorylation of ERK, under which vemurafenib synergistically enhanced the DHT‐augmented phosphorylation. The enhanced ERK phosphorylation was no longer detectable by adding an ERK inhibitor, U0126. On the other hand, as mTOR plays an important role in the regulation of sebum production, the phosphorylation of Akt and 4EBP1, which are the upstream and downstream molecules in mTOR signalling, respectively, was increased in the DHT‐treated hamster sebocytes. Vemurafenib inhibited the DHT‐augmented 4EBP1 phosphorylation, which was no longer detectable in the presence of U0126. Furthermore, the suppression of the DHT‐augmented sebum production and accumulation by vemurafenib was restored to their levels in DHT alone upon the U0126 treatment. Thus, these results provide novel evidence that vemurafenib suppresses sebum production and accumulation by the vemurafenib‐activated ERK signalling that inhibits the Akt/mTOR pathway in DHT‐differentiated hamster sebocytes.

## Graphical abstract

Vemurafenib suppresses sebum production in DHT‐differentiated hamster sebocytes by inducing paradoxical ERK activation, leading to the inhibition of the Akt/mTOR pathway.

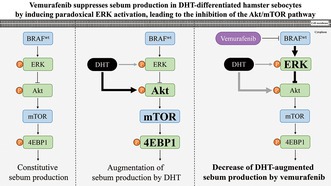

## 1 Introduction

Cutaneous melanoma is a malignant and metastatic tumour with an oncogenic mutation V600E in B‐Raf proto‐oncogene serine/threonine kinase (BRAF). The genetic mutation enhances downstream mitogen‐activated kinase (MAPK)/extracellular signal‐regulated kinase (ERK) signalling by continuously activating BRAF [1]. A low‐molecular‐weight BRAF inhibitor, vemurafenib, has been shown to inhibit MAPK signalling, specifically in melanoma harbouring the BRAF^V600E^ mutation, resulting in an antiproliferative action against malignant tumour cells in individuals diagnosed with cutaneous melanoma [2]. While the efficacy of vemurafenib in the treatment of melanoma is highly regarded, cutaneous side effects, including dry skin, have been reported to occur in 10%–40% of patients who undertook the targeted agents including vemurafenib [3]. Unexpected and undesirable cutaneous manifestations not only reduce patients' quality of life (QOL) and cause aesthetic distress but also decrease their motivation to undergo cancer treatment. The alleviation and reduction of such adverse symptoms may lead to improved outcomes of anticancer pharmacotherapy.

The sebaceous gland is an integral part of the pilosebaceous unit (PSU), which is composed of the hair, hair follicle, and arrector pili muscle [4]. Sebaceous gland cells (sebocytes) synthesise sebum, of which a main component is triacylglycerol, with wax esters and squalene as other components [5, 6, 7, 8, 9]. Sebum production has been reported to be augmented by 5α‐dihydrotestosterone (DHT) and insulin in sebocytes from humans and rodents [10]. Sebum secreted on the skin surface forms a biological lipid barrier that is responsible for sustaining moisture in the skin [11]. Disorder of the sebaceous glands is likely to result in the dysregulation of sebum production and secretion, leading to skin dryness symptoms from inadequate cutaneous barrier formation. For example, the reduced lipid content on the skin surface of atopic dermatitis patients has been reported to contribute to skin dryness and itching [12] However, it is unclear whether vemurafenib‐induced skin dryness is associated with abnormal sebaceous gland functions.

In the present study, we examined the effect of vemurafenib on the production of sebum in hamster sebocytes and demonstrated that vemurafenib decreased serum production and accumulation in DHT‐differentiated hamster sebocytes. In addition, the vemurafenib‐suppressed sebum production resulted from an increase in extracellular signal‐regulated kinase (ERK) phosphorylation and the sequential inhibition of the mechanistic target of rapamycin (mTOR) pathways in hamster sebocytes.

## 2 Methods

### 2.1 Cell Culture and Treatment

Hamster sebocytes (2.35 × 10^4^ cells per cm^2^) were cultured in D‐MEM/Ham's F‐12 (1:1) with L‐glutamine and phenol red (FUJIFILM Wako Pure Chemical, Osaka, Japan) supplemented with 6% heat‐inactivated fetal bovine serum (FBS) (Sigma‐Aldrich Japan, Tokyo, Japan), 2% human serum (ICN Biochemicals, Costa Mesa, CA, USA), and recombinant human epidermal growth factor (10 ng per mL) (Progen Biotechnik GmbH, Heidelberg, Germany) to achieve complete cell adhesion. The cells were treated every 2 days for up to 7 days with or without vemurafenib (0.1–10 μM) (Selleck Chemicals Japan, Osaka, Japan) in the presence or absence of DHT (100 μM) (Sigma‐Aldrich, St. Louis, MO, USA) or insulin (10 nM) (Sigma‐Aldrich) as previously described [13, 14]. In this series of experiments, the cells were used up to the 5th passage.

### 2.2 Analysis of Sebum Production by Nile Red and Calcein‐AM Staining

The cells were stained with nile red (5.0 μM) (Sigma Chemical, St. Louis, MO, USA) at 37°C for 30 min. The fluorescent intensity of the Nile red‐stained cells was measured by a multimode microplate reader, SpectraMax iD3 (Molecular Devices, San Jose, CA, USA), at 485 nm (excitation) and 565 nm (emission). After that, the cells were stained with calcein‐AM (0.5 mg/mL) (Dojindo Laboratories, Kumamoto, Japan) at 37°C for 30 min. The fluorescent intensity of the calcein‐AM‐administrated cells, which was used as an indicator of viable cells [15], was measured by SpectraMax iD3 at 475 nm (excitation) and 515 nm (emission). The sebum production was measured by calculating the ratio of the fluorescence intensity of Nile red against calcein. This ratio was then represented as the mean value of the control, either as a relative value or 100%.

### 2.3 Oil Red O Staining

After treating the sebocytes with DHT, the cells were washed once with Ca^2+^‐ and Mg^2+^‐free phosphate‐buffered saline [PBS (−)] and fixed with 4% paraformaldehyde (FUJIFILM Wako Pure Chemical) diluted with PBS (−) for 30 min at room temperature. The cells were washed with distilled H_2_O and then stained with 0.3% oil‐red O (Sigma Chemical) in isopropanol: distilled H_2_O (3:2, vol:vol) at 37°C for 30 min. The stained cells were washed with PBS (−) and then viewed with a light microscope furnished with a digital camera (Olympus Optical, Tokyo, Japan).

### 2.4 Quantitative Real‐Time PCR

The gene expressions of stearoyl‐CoA desaturase‐1 (SCD‐1), diacylglycerol acyltransferase 1 (DGAT‐1), and perilipin‐1 (PLIN‐1) were analysed by a quantitative real‐time PCR as previously reported [16]. Total RNA was isolated from the cells using ISOGEN II (Nippon Gene, Toyama, Japan), and then an aliquot of RNA (500 ng) was subjected to a reverse transcriptase reaction for the synthesis of cDNA using a PrimeScript RT reagent (Takara Bio, Shiga, Japan) according to the manufacturer's instructions. Aliquots (an equivalent of 2.5 ng of total RNA) of the transcript were subjected to real‐time PCR for SCD‐1, DGAT‐1, PLIN‐1, and glyceraldehyde‐3‐phosphate dehydrogenase (GAPDH) mRNAs using THUNDERBRID SYBR qPCR Mix (Toyobo, Osaka, Japan) and their specific primers (Table S1). The amplification cycle was performed at 94°C for 5 s and 60°C for 30 s using a Thermal Cycler Dice Real Time System TP‐800 (Takara Bio). The obtained threshold cycle (CT) value for SCD‐1, DGAT‐1, and PLIN‐1 was normalised by that for GAPDH, and the relative expression level was expressed as the mean value of the control as 1.

### 2.5 Western Blotting

The harvested cell lysate (10 μg protein) was subjected to Western blot analysis using a 12.5% acrylamide gel. The membrane was reacted with Phospho‐4E‐BP1 (Thr37/46) (236B4) Rabbit mAb, 4E‐BP1 (53H11) Rabbit mAb, Phospho‐Akt (Ser473) (D9E) XP Rabbit mAb, Akt (pan) (C67E7) Rabbit mAb, Phospho‐p44/42 MAPK (Erk1/2) (Thr202/Tyr204) (D13.14.4E) XP Rabbit mAb, and p44/42 MAPK (Erk1/2) (137F5) Rabbit mAb (Cell Signalling Technology, Danvers, MA, USA). Immunoreactive phosphorylated 4EBP1 (p4EBP1), total 4EBP1, phosphorylated ERK (pERK), total ERK, phosphorylated Akt (pAkt), and total Akt were visualised with ImmunoStar LD (FUJIFILM Wako Pure Chemical) according to the manufacturer's instructions. Relative signal intensity of the p4EBP1 protein against total 4EBP1, pERK against total ERK, and pAkt against total Akt was quantified by densitometric scanning using a FUSION‐Chemiluminescence Imaging System (Vilber Bio Imaging, Collégien, France), and the relative expression level was expressed as the mean value of the control as 1.

### 2.6 Statistical Analysis

Statistical analyses were performed using a one‐way ANOVA with the Tukey–Kramer test or Fisher's Least Significant Difference test adjusted by Dunnett's test for multiple comparisons. *p*‐values less than 0.05 were considered statistically significant.

## 3 Results

### 3.1 Suppression of Sebum Accumulation and Production by Vemurafenib in DHT‐Differentiated Hamster Sebocytes

**FIGURE 1:**
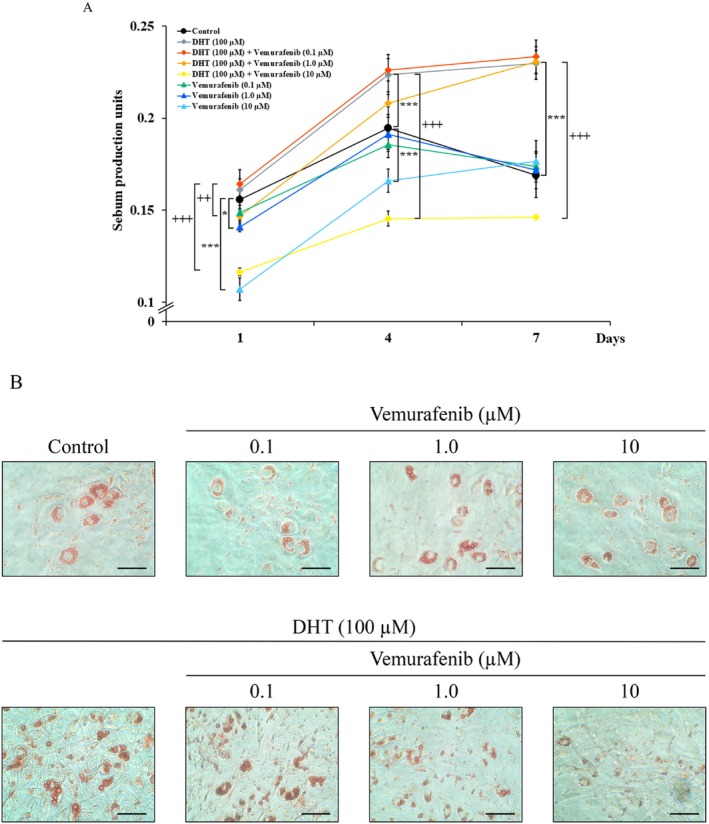
Suppression of sebum production and accumulation by vemurafenib in DHT‐differentiated hamster sebocytes. Hamster sebocytes at the third passage were treated every 2 days for up to 7 days with or without vemurafenib (0.1–10 μM) in the presence of DHT (100 μM), and then the intracellular level of sebum (A) and lipid‐droplet formation (B) were analysed by Nile Red and Oil Red O staining, respectively, as described in the METHODS. Four independent experiments were reproducible, and typical findings are shown as mean ± SD. * and ***, significantly different from the untreated cells (Control) (*p* < 0.05 and 0.001, respectively). ++ and +++, significantly different from the DHT‐treated cells (DHT) (*p* < 0.01 and 0.001, respectively). Scale bars = 10 μm.

Given that sebocytes treated with DHT produce and store sebum as intracellular lipid droplets [17, 18, 19, 20], we first examined the effect of vemurafenib on sebum production and accumulation in DHT‐differentiated hamster sebocytes. DHT (100 μM) augmented sebum production for up to 7 days in a time‐dependent manner (Figure 1A). In addition, the increased sebum accumulation was detectable in the DHT‐treated cells (Figure 1B). When hamster sebocytes were treated with vemurafenib (10 μM) in the presence of DHT, the DHT‐augmented sebum production was decreased by vemurafenib in a dose‐dependent manner (Figure 1A, 49.5% ± 5.5% inhibition at 10 μM at the 7 days; *p* < 0.001). In addition, the DHT‐augmented intensity of oil red O staining decreased in the vemurafenib‐treated cells (Figure 1B). Furthermore, there was no change in cell viability in hamster sebocytes under these conditions (Figure S1). Interestingly, another sebogenesis factor, insulin‐induced sebum production and accumulation was not influenced by vemurafenib in the hamster sebocytes (Figure S2). Therefore, these results show that vemurafenib preferentially had an inhibitory effect on DHT‐dependent sebum production and its accumulation in hamster sebocytes.

### 3.2 Suppression of the Expression of SCD‐1, DGAT‐1, and PLIN‐1 mRNA by Vemurafenib in DHT‐Differentiated Hamster Sebocytes

**FIGURE 2:**
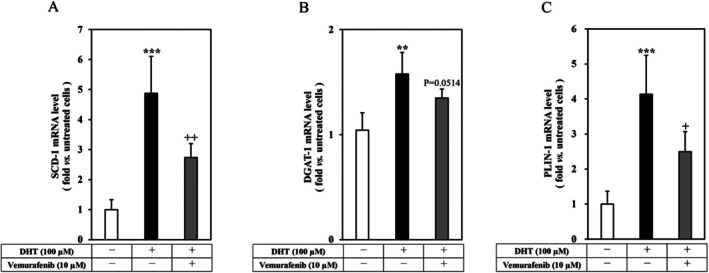
Effects of vemurafenib on the gene expression of SCD‐1, DGAT‐1, and PLIN‐1 in DHT‐differentiated hamster sebocytes. Hamster sebocytes at the third passage were treated every 2 days for up to 7 days with or without vemurafenib (10 μM) in the presence of DHT (100 μM). The harvested RNA was subjected to RT‐PCR for SCD‐1 (A), DGAT‐1 (B), and PLIN‐1 (C) as described in the Methods. Four independent experiments were reproducible, and typical findings are shown as mean ± SD. ** and ***, significantly different from the untreated cells (*p* < 0.01 and 0.001, respectively). + and ++, significantly different from the DHT‐treated cells (*p* < 0.05 and 0.01, respectively).

We have reported that the predominant lipid component of hamster sebocyte‐derived sebum is triacylglycerol (TG), whereas free fatty acids and wax esters were found to be minor components [20]. SCD‐1 has been reported to be a crucial regulator of maintaining sebum because it catalyses the production of monounsaturated fatty acids such as TG in sebaceous glands [21]. DGAT‐1 is a rate‐limiting enzyme in TG synthesis [22], and is characterised as a sebocyte‐specific subtype in hamsters [14]. PLIN‐1, a member of the PAT family, is located on the surface of lipid droplets and participates in lipid droplet formation and lipolysis [23, 24]. To clarify the regulatory mechanism of sebum production and accumulation by vemurafenib, we examined the regulation of SCD‐1, DGAT‐1, and PLIN‐1 gene expression by vemurafenib in the DHT‐treated hamster sebocytes. As shown in Figure 2, DHT (100 μM) augmented the gene expressions of SCD‐1 (panel A), DGAT‐1 (panel B), and PLIN‐1 (panel C) in the hamster sebocytes. In addition, the augmented gene expressions of SCD‐1 and PLIN‐1 were significantly decreased, while that of DGAT‐1 was slightly suppressed by vemurafenib (10 μM). Therefore, these results indicated that vemurafenib transcriptionally suppressed the expressions of SCD‐1, DGAT‐1, and PLIN‐1 in the DHT‐differentiated hamster sebocytes.

### 3.3 Activation of ERK by Vemurafenib in DHT‐Differentiated Hamster Sebocytes

**FIGURE 3:**
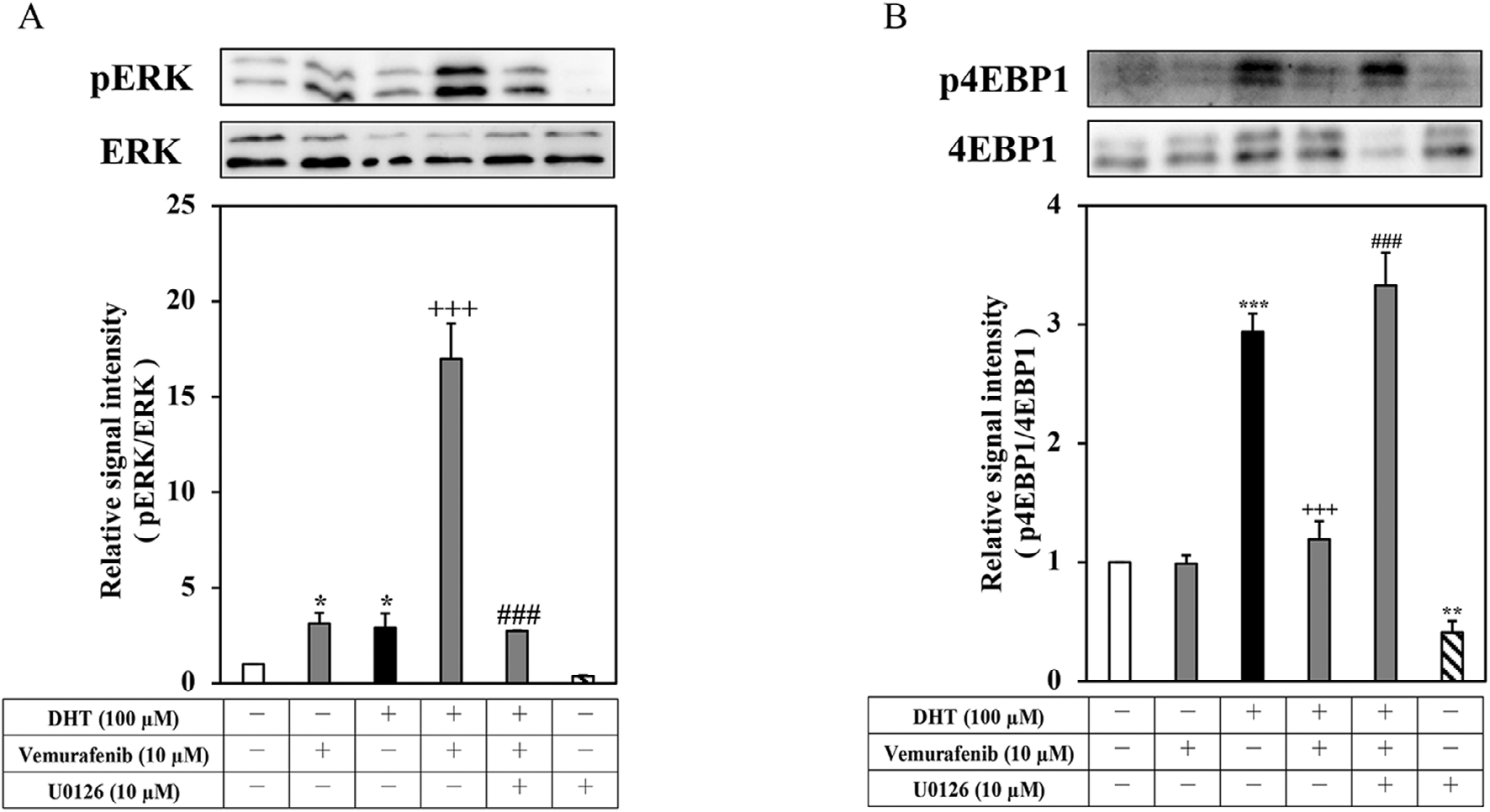
Regulation of ERK and 4EBP1 phosphorylation by vemurafenib in DHT‐differentiated hamster sebocytes. Hamster sebocytes at the third passage were treated every 2 days for up to 7 days with or without DHT (100 μM) in the presence of vemurafenib (10 μM) and/or U0126 (10 μM), and then pERK (A) and p4EBP1 (B) were analysed as described in the METHODS. Three independent experiments were reproducible, and typical findings are shown as mean ± SD. *, **, and ***, significantly different from the untreated cells (*p* < 0.05, 0.01, and 0.001, respectively). ^+++^, significantly different from the DHT‐treated cells (*p* < 0.001). ^###^, significantly different from the DHT and vemurafenib‐treated cells (*p* < 0.001).

Although BRAF inhibitors inhibit the MEK/ERK pathway in melanoma with the BRAF^V600E^ mutation, it has been reported that a paradoxical activation of the MAPK pathway in BRAF wild‐type cells is linked to the adverse actions of vemurafenib [25, 26, 27]. Therefore, we examined the regulation of ERK phosphorylation by vemurafenib in DHT‐differentiated hamster sebocytes. As shown in Figure 3A, vemurafenib (10 μM) and DHT (100 μM) were found to augment the phosphorylation of ERK in sebocytes. In addition, the co‐treatment of vemurafenib and DHT synergistically enhanced ERK phosphorylation. Furthermore, the enhancement of ERK phosphorylation was no longer detectable in the presence of an ERK inhibitor, U0126 (10 μM). Thus, these results indicate that vemurafenib promoted the phosphorylation of ERK in DHT‐differentiated hamster sebocytes.

### 3.4 Involvement of the Enhanced ERK Phosphorylation in the Suppression of Sebum Production and Accumulation by Vemurafenib in DHT‐Differentiated Hamster Sebocytes

**FIGURE 4:**
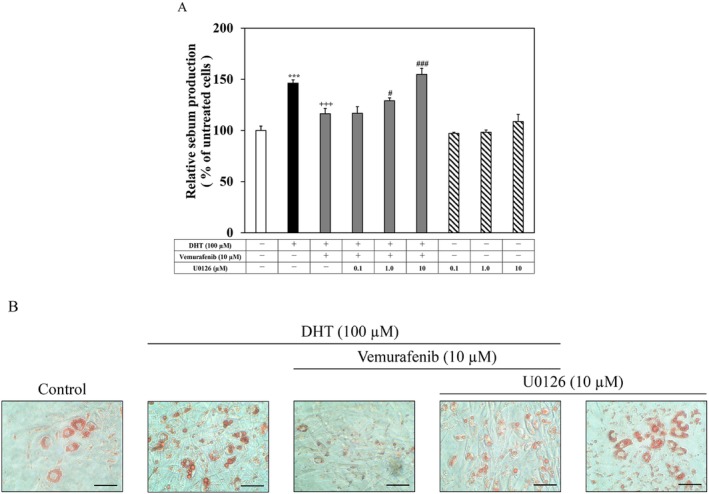
Involvement of the ERK signalling in vemurafenib suppression of sebum production and accumulation in DHT‐differentiated hamster sebocytes. Hamster sebocytes at the third passage were treated every 2 days for up to 7 days with or without DHT (100 μM) and vemurafenib (10 μM) in the presence or absence of U0126 (0.1–10 μM), and then the intracellular level of sebum (A) and lipid‐droplet formation (B) were analysed as shown in Figure 1. Four independent experiments were reproducible, and typical findings are shown as mean ± SD. (A): ***, significantly different from the untreated cells (*p* < 0.001). +++, significantly different from the DHT‐treated cells (*p* < 0.001). # and ###, significantly different from the cells treated with DHT and vemurafenib (*p* < 0.05 and 0.001, respectively). (B): scale bars =10 μm.

Next, we examined whether the enhanced ERK phosphorylation was associated with the suppression of sebum production and accumulation by vemurafenib in DHT‐differentiated hamster sebocytes. As shown in Figure 4, when hamster sebocytes were treated with DHT and vemurafenib in the presence of U0126, the reduced sebum levels were restored to the levels of DHT alone when treated with U0126 in a dose‐dependent manner, whereas there was no change in the constitutive sebum level in the U0126‐treated cells. In addition, similar phenomena were detectable in the sebum accumulation (Figure 4B). Therefore, these results show that the enhanced phosphorylation of ERK by vemurafenib resulted in a decrease in sebum production and accumulation in the DHT‐differentiated hamster sebocytes.

### 3.5 Inhibition of the mTOR Pathway by Vemurafenib Through the Activation of the ERK Pathway in DHT‐Differentiated Hamster Sebocytes

Mechanistic target of rapamycin (mTOR), a major regulator of cellular protein and lipid metabolism, has been reported to increase in the sebaceous glands of acne patients [28, 29]. Since the ERK pathway has been reported to stimulate the mTOR pathway by controlling mTORC1 [30], we examined whether vemurafenib influenced the activation of the mTOR pathway in DHT‐differentiated hamster sebocytes. As shown in Figure 3B, the phosphorylation of 4EBP1, which is a downstream molecule in mTOR signalling [31], was augmented by DHT (100 μM) in the hamster sebocytes. Vemurafenib was found to decrease the phosphorylation of 4EBP1 in the DHT‐differentiated cells. In addition, the inhibition of 4EBP1 phosphorylation by vemurafenib was no longer detectable by adding U0126, while the constitutive phosphorylation of 4EBP1 was slightly decreased by U0126 alone. Furthermore, vemurafenib was found to suppress the phosphorylation of Akt, which is an upstream signal mediator in mTOR signalling, in the DHT‐treated cells (Figure S3). Therefore, these results show that vemurafenib inhibited the Akt/mTOR signal pathway through the activation of ERK signalling in DHT‐differentiated hamster sebocytes.

## 4 Discussion

Vemurafenib is a kinase inhibitor specifically designed to target tumours harbouring the BRAF^V600E^ mutation and has considerable potential as a therapeutic intervention for patients with unresectable or metastatic melanoma. In addition, the side effects of vemurafenib have been reported to include dry skin, acneiform eruption, and seborrheic keratosis. Among such side effects, dry skin symptoms have been reported to appear in 10%–40% of patients who undertook targeted agents including vemurafenib [3]. Recently, Yin H et al. have reported that skin dryness in patients with atopic dermatitis is caused by changes in sebum composition related to the dysregulation of lipid metabolism in sebaceous glands [12]. In the present study, we demonstrated for the first time that vemurafenib decreased sebum production in the DHT‐differentiated hamster sebocytes but did not influence the constitutive sebum production. In addition, the DHT‐augmentation of the oil‐red O‐positive cell number was no longer detectable in the vemurafenib‐treated cells, whereas there was no change in the intracellular accumulation of sebum in sebocytes treated with vemurafenib alone. Therefore, these results suggest that vemurafenib inhibits sebum production and accumulation in sebaceous glands, leading to an understanding of the pathogenesis of skin dryness, which is associated with the inhibition of sebum production [32], at least among the vemurafenib‐induced side effects.

Several important regulatory factors are known to be involved in sebogenesis and intracellular accumulation of sebum in humans and rodents. Since two isoforms of SCD have been reported to be characterised in humans and four in mice [33], SCD is responsible for the synthesis of monounsaturated fatty acids by catalysing the introduction of a *cis* double bond at the *Δ*9 position of saturated acyl‐CoA molecules containing either 16 or 18 carbon atoms [34]. In addition, SCD‐1 has been reported to play an important role in the synthesis of fatty acids in sebocytes in humans and hamsters [35, 36, 37, 38]. The rate‐limiting enzyme of TG production is known as DGAT, of which two isozymes are DGAT‐1 and DGAT‐2 [39, 40]. DGAT‐1 rather than DGAT‐2 has been reported to be involved in TG production in mammals [41, 42]. Regarding sebum accumulation, PLIN‐1/perilipin A has been reported to be localised on the surface of intracellular lipid droplets in the differentiated hamster sebocytes [24]. On the other hand, it has been reported that the functional integrity of sebaceous glands is disrupted in SCD‐1‐deficient mice in the regulation of lipid metabolism and cell differentiation [43, 44]. Furthermore, Fluhr JW et al. have reported that the decreased stratum corneum hydration in SCD‐1‐deficient mice is due to reduced glycerol content resulting from the diminished sebaceous gland‐derived TG hydrolysis [45]. In this study, we demonstrated that vemurafenib significantly suppressed the gene expression of SCD‐1 and PLIN‐1, and slightly that of DGAT‐1 in the DHT‐differentiated hamster sebocytes. In addition, the production of PLIN‐1 has been reported to be closely associated with sebum accumulation in sebocytes from humans and hamsters [24, 42, 46, 47]. Therefore, vemurafenib‐induced suppression of sebum production and accumulation is likely to result from the transcriptional inhibition of SCD‐1, DGAT‐1, and PLIN‐1 expression in the DHT‐differentiated hamster sebocytes, which may partially contribute to the onset of dry skin among patients treated with vemurafenib.

Sebum production and cell proliferation in sebaceous glands have been reported to be controlled in an androgen‐dependent manner [48]. Excess production of androgens or attenuated androgen signalling with aging has been shown to be associated with puberty, acne, and senile xerosis, respectively [49, 50]. On the other hand, Caroppo F et al. have reported on the epidemiological link between a longer cumulative exposure to sex hormones, such as androgens, and the risk of melanoma [51]. In addition, it has been reported that androgens such as testosterone and DHT are responsible for activating the androgen receptor‐dependent MAPK/ERK signalling pathway [52]. Taken together with our findings that vemurafenib‐mediated suppression of sebum production and accumulation was detectable in hamster sebocytes treated with DHT but not insulin, vemurafenib may inhibit the DHT‐induced MAPK/ERK signalling pathway in sebocytes. Unexpectedly, in the present study, although DHT increased the phosphorylation of ERK, vemurafenib promoted the phosphorylation of ERK in hamster sebocytes. Furthermore, the co‐treatment with vemurafenib and DHT synergistically enhanced ERK phosphorylation, which was no longer detectable in the presence of U0126, suggesting that vemurafenib facilitated the activation of ERK signalling in sebocytes. These findings are supported by a recent report of Corrales E et al. that vemurafenib paradoxically activates ERK signalling in human dermal fibroblasts [53]. Moreover, it has been reported that BRAF inhibitors paradoxically activate the ERK signalling pathway in cells with wild‐type BRAF heterodimerised with CRAF [54, 55]. Taken together with our preliminary experiment that hamster sebocytes express the wild‐type of the *Braf* gene (data not shown), it is suggested that the paradoxical activation of ERK by vemurafenib is involved in the suppression of the DHT‐induced sebum production in hamster sebocytes. Further investigation is needed to clarify the molecular mechanisms of the vemurafenib‐induced paradoxical ERK activation for the regulation of sebum production in hamster sebocytes.

Mechanistic target of rapamycin plays a crucial role as a sensor for various signals from both intracellular and extracellular sources such as growth hormones, amino acids, and stress for controlling cell proliferation and metabolic regulation [56]. mTOR has been reported to be categorised into two distinct complexes, namely mTOR complex 1 (mTORC1) and mTOR complex 2 (mTORC2). The activation of mTORC1 has been reported to be involved in lipid synthesis and sebaceous TG production [29, 38]. On the other hand, ERK signalling has been reported to extensively participate in cell survival, migration, and motility through positive and negative crosstalk with mTORC1 signalling [30]. In addition, it has been reported that ERK phosphorylates Raptor, an essential scaffolding protein of mTORC1, to promote Ras‐dependent mTORC1 activation through the phosphorylation of 4EBP1 [57]. In the present study, we found for the first time that the paradoxical activation of ERK signalling by vemurafenib completely suppressed the DHT‐induced phosphorylation of 4EBP1 in hamster sebocytes, which was no longer detectable in the presence of U0126. These findings were supported by a previous report where the expression of 4EBP1 was negatively regulated at the transcriptional level by the activation of ERK via the induction of Egr‐1 expression [58]. Furthermore, vemurafenib decreased the phosphorylation of Akt, which is an upstream signal mediator of mTOR [56], in the DHT‐treated hamster sebocytes. Thus, these results strongly suggest that the activation of ERK signalling negatively regulates the Akt/mTORC1 signalling pathway in sebocytes. Furthermore, the ERK‐dependent inhibition of the Akt/mTORC1 pathway is likely to contribute to the suppression of sebum production and accumulation by vemurafenib in DHT‐differentiated hamster sebocytes.

In conclusion, we demonstrated that vemurafenib suppressed the production and intracellular accumulation of sebum in hamster sebocytes differentiated with DHT, but not insulin. In addition, the vemurafenib‐mediated decrease of sebum production and accumulation resulted from the inhibition of SCD‐1, DGAT‐1, and PLIN‐1 expression in the DHT‐differentiated cells. Furthermore, vemurafenib paradoxically enhanced the phosphorylation of ERK in the DHT‐differentiated cells, by which 4EBP1 phosphorylation was inhibited to suppress sebum production. Thus, these results provide novel evidence that vemurafenib suppresses sebum production and accumulation by the paradoxical activation of BRAF/MEK/ERK signalling that inhibits the Akt/mTORC1 pathway in DHT‐differentiated hamster sebocytes. Our findings are likely to help in understanding the mechanism of skin disorders, at least dry skin, as a side effect caused by vemurafenib.

## Author Contributions

Conceptualization: T.K. and T.S.; experiment execution: T.K.; statistical analysis: T.K.; methodology: T.K. and T.S.; equipment development and operation: T.K. and T.S.; project administration: T.S.; supervision: T.S.; writing – review and editing: T.K. and T.S. All authors read and approved the final manuscript.

## Conflicts of Interest

The authors declare no conflicts of interest.

## Supporting information


**Appendix S1:** exd70150‐sup‐0001‐AppendixS1.docx.

## Data Availability

The data that support the findings of this study are available from the corresponding author upon reasonable request.
