# Supplementary material for: Suppression of Sebum Production by Vemurafenib Through Paradoxical ERK Activation Resulted in the Inhibition of the mTOR Pathway in 5α‐Dihydrotestosterone‐Differentiated Hamster Sebocytes In Vitro
Source: Exp Dermatol. 2025 Aug 16;34(8):e70150. doi: 10.1111/exd.70150 (PMC12357165; doi:10.1111/exd.70150)
Supplement: Supplementary file 1 — Appendix S1: exd70150‐sup‐0001‐AppendixS1.docx. [file EXD-34-e70150-s001.docx]

**Supplemental Table and Figures**

**Suppression of sebum production by vemurafenib through paradoxical ERK activation resulted in the inhibition of mTOR pathway in 5α-dihydrotestosterone-differentiated hamster sebocytes *in vitro***

Toshikazu Koiwai and Takashi Sato^*^

Department of Biochemistry, Tokyo University of Pharmacy and Life Sciences, Tokyo, Japan

^*^*Correspondence author*: Takashi Sato, PhD

Department of Biochemistry, School of Pharmacy, Tokyo University of Pharmacy and Life Sciences, 1432-1 Horinouchi, Hachioji, Tokyo 192-0392, Japan,

Tel.: +81-42-676-5706

Fax: +81-42-676-5734

E-mail: satotak@toyaku.ac.jp

Table S1 Primer sequences of hamster SCD-1, DGAT-1, PLIN-1, and GAPDH


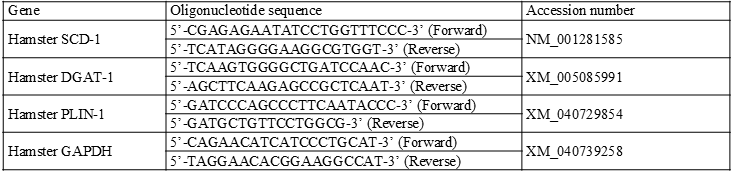


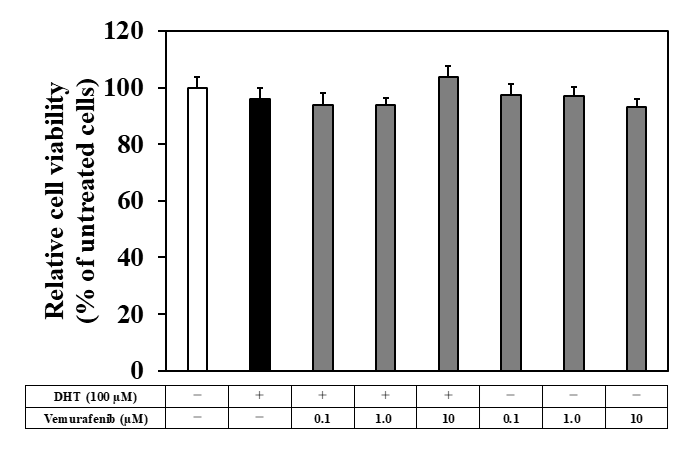


Figure S1 Effects of vemurafenib on cell viability in DHT-differentiated hamster sebocytes

Hamster sebocytes at the 3rd passage were treated every two days for seven days with or without vemurafenib (0.1–10 μM) in the presence or absence of DHT (100 µM) and then cell viability was analyzed by calcein-AM staining as described in the METHODS. Four independent experiments were reproducible and typical findings are shown. The relative cell viability is indicated as mean ± SD.


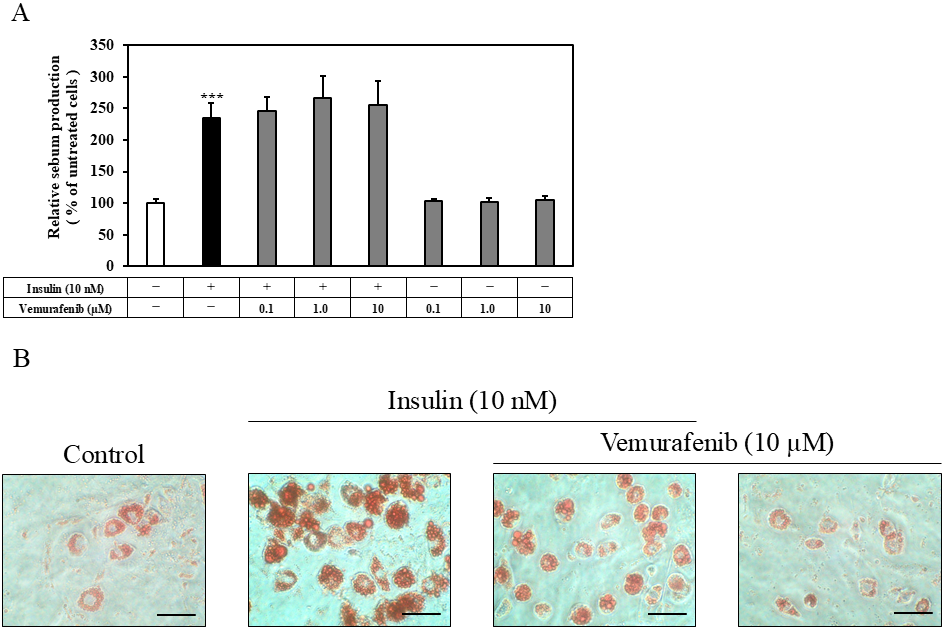


Figure S2 Effects of vemurafenib on sebum production and accumulation in insulin-differentiated hamster sebocytes

Hamster sebocytes at the 3rd passage were treated every two days for seven days with or without vemurafenib (0.1–10 μM) in the presence or absence of insulin (10 nM) and then the intracellular level of sebum (A) and lipid-droplet formation (B) were analyzed by nile red and oil red O staining, respectively, as described in the METHODS. Four independent experiments were reproducible, and typical findings are shown. The relative sebum production is indicated as mean ± SD. ^***^, significantly different from the untreated cells (Control) (p<0.001). Scale bars =10 μm.


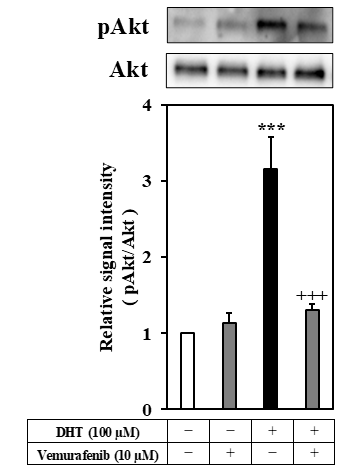


Figure S3 Suppression of DHT-augmented Akt phosphorylation by vemurafenib in hamster sebocytes

Hamster sebocytes at the 3rd passage were treated every two days for seven days with DHT (100 μM) and/or vemurafenib (10 μM) and then phosphorylated Akt (pAkt) and Akt were analyzed as described in the METHODS. Three independent experiments were reproducible and typical findings are shown. The relative level of pAkt against total Akt is indicated as mean ± SD. ^***^, significantly different from the untreated cells (p<0.001). ^+++^, significantly different from the DHT-treated cells (p<0.001).
